# A Manual of Dental Anatomy, Human and Comparative

**Published:** 1882-06

**Authors:** 


### EDITORIAL.

### A MANUAL OF DENTAL ANATOMY, HUMAN AND COMPARATIVE.

BY CHAS. S. TOMES, M.A F.R.S.

*With* 191 *Illustrations—Second Edition.*

This, the second edition, following so soon the first, shows the popularity and high esteem in which the work is held by those interested in the subject, upon which it treats.

The general verdict, in regard to the first edition, was, well done, so far as it extended, and could not be easily improved, still the author has thought best to make a thorough revision. Some of the matter has been largely re-written. The author, in the preface, says: "Some portions, notably the chapter on the dental tissues, have been in great part re-written, and many new illustrations have been added here and elsewhere in the book."

This work fills a want that has long existed. Dentists have felt the need of such a work; and the students of anatomy every where will highly appreciate, and derive great assistance from this Manual.

Mr. Tomes recognized ability gives assurance for the correctness of the work; and the facilities at his command have enabled him to bring in this condensed form a large amount of valuable material. No dentist or student of anatomy should be without this work.

It is issued by Priestly Blakiston & Sons, of Philadelphia, which is sufficient guarantee for the style of the book. It may be obtained of Robt. Clarke, of this city, and of book-sellers generally. Price, $4.25.

---

The Kentucky State Dental Association will hold its annual meeting in Louisville, commencing Tuesday, June 6, 1882, at the rooms of the Polytechnic Society. Dentists of Kentucky, and neighboring States, are cordially invited to attend.

CHAS. E. DUNN.

### FOSSILINE.

Of the white plastic filling materials—oxyphosphates and oxychlorides—none which I have ever used have possessed more desirable qualities than is shown, and in some respect proven by *fossiline.* It has been tested for about three years, and thus far gives excellent results. It sets readily, though not too quickly, and becomes very hard in a short time. It hardens about as promptly and as perfectly in saliva as when free from moisture. It will be well, however, to keep it dry till finished and the surface oiled. I have used it about nine months and have in no case found it affected by the secretions of the mouth. It is certainly worthy the attention of all who are seeking the best things.

